# A 300-GHz low-cost high-gain fully metallic Fabry–Perot cavity antenna for 6G terahertz wireless communications

**DOI:** 10.1038/s41598-021-87076-3

**Published:** 2021-04-08

**Authors:** Basem Aqlan, Mohamed Himdi, Hamsakutty Vettikalladi, Laurent Le-Coq

**Affiliations:** 1 Institut d’Electronique et des Technologies du numéRique (IETR), UMR CNRS 6164, Université de Rennes 1, Campus de Beaulieu, 35042 Rennes Cedex, France; 2 Department of Electrical Engineering, King Saud University, Riyadh, 11421 Saudi Arabia

**Keywords:** Engineering, Electrical and electronic engineering

## Abstract

A low-cost, compact, and high gain Fabry–Perot cavity (FPC) antenna which operates at 300 GHz is presented. The antenna is fabricated using laser-cutting brass technology. The proposed antenna consists of seven metallic layers; a ground layer, an integrated stepped horn element (three-layers), a coupling layer, a cavity layer, and an aperture-frequency selective surface (FSS) layer. The proposed aperture-FSS function acts as a partially reflective surface, contributing to a directive beam radiation. For verification, the proposed sub-terahertz (THz) FPC antenna prototype was developed, fabricated, and measured. The proposed antenna has a measured reflection coefficient below − 10 dB from 282 to 304 GHz with a bandwidth of 22 GHz. The maximum measured gain observed is 17.7 dBi at 289 GHz, and the gain is higher than 14.4 dBi from 285 to 310 GHz. The measured radiation pattern shows a highly directive pattern with a cross-polarization level below − 25 dB over the whole band in all cut planes, which confirms with the simulation results. The proposed antenna has a compact size, low fabrication cost, high gain, and wide operating bandwidth. The total height of the antenna is 1.24 ${\lambda }_{0}$ (${\lambda }_{0}$ at the design frequency, 300 GHz) , with a size of 2.6 mm × 2.6 mm. The proposed sub-THz waveguide-fed FPC antenna is suitable for 6G wireless communication systems.

## Introduction

High transmission data rates, low latency, high reliability and interference-free operation are the most needed features today for applications ranging from communications to infotainment and positioning to healthcare. The demand is the driving force behind recent remarkable developments in wireless networks in new frequency^1^. This leads to making the terahertz (THz) (0.1–10 THz) band the key candidate for future wireless networks. Successful deployment of wireless networks relies heavily on antenna design, and future wireless networks are no exception. Recently, a new IEEE standard (802.15.3d-2017) has been established around 300 GHz, and data rates transmissions up-to 100 Gb/s have already been demonstrated^2^. The sub-THz band (0.1–1 THz) around 300 GHz could be key enablers as strong candidate technology to realize sixth generation (6G) wireless communication speeds up to 1 Tpbs^1,3^. Moving up to sub-THz range frequency means a drastic increase in free-space path-loss and atmospheric absorption (i.e., absorption by molecules in air). Consequently, unprecedentedly high gain antennas are necessary to compensate for the path-loss. Antenna arrays with complex feeding networks can provide better directivity and gain but have narrow bandwidth and high loss especially at high THz frequencies due to feeding, and substrate materials losses, which has a great influence on the performance of antennas resulting in a significant decrease in antenna radiation efficiency. Horn antennas^4,5^ and reflector antennas^6^ have been reported with good radiation patterns, low cross-polarization and wide-band operation, but have the shortcoming of bulky size, which makes them difficult to assemble with planar circuits. Lens antennas have also been proposed at higher THz frequencies as good directivity and high gain antennas for broadside radiation^7–9^. The antenna-based lens normally has a profile of several wavelengths, which is increased even more if a higher gain value is desired, and can be challenging at the sub-THz band (i.e. 300 GHz) in terms of compact integrated systems. Another serious weakness of dielectric lens antennas is the surface wave effect and dielectric loss, and it is necessary to optimize its material and geometry in the future. Contrarily, Fabry–Perot cavity (FPC) antennas featuring high radiating performance and low profile and low cost and low complexity of fabrication, have attracted broad interests from research in recent years. However, most FPC antennas reported to date have been extensively investigated in the microwave range^10–12^ and recently in the millimeter-wave (MMW) band by applying single-layer frequency selective surface (FSS) as a partially reflective surface (PRS)^13,14^. Authors in^15,16^ have successfully proposed the FPC antenna designs at THz band. Nevertheless, due to the limitation of fabrication technologies, the first steps towards experimental validation of FPC antennas have already been taken in^17^ by using a SU-8 photoresist micromachining technology at 284 GHz. Although this design has been fabricated, it cannot provide a wide bandwidth (~ 4.5 GHz) and the measured results of radiation patterns are not up to the mark.

In this work, we will introduce a fully metallic FPC antenna design that operates at 300 GHz and is fabricated with laser-cutting brass technology with good accuracy at such high frequency. The proposed antenna consists of seven metallic brass layers arranged from the bottom to the top as following, a ground layer, an integrated stepped horn element (three-layers), coupling layer, cavity layer, and an aperture-FSS layer. The whole antenna structure’s feeding is done by using a standard WM-864 rectangular waveguide with UG-387/U flange, which provides a frequency range between 220 and 330 GHz. The proposed aperture-FSS layer functions as a partially reflective surface, contributing to a high directive gain for the antenna. For realization, the complete antenna structure, including the standard UG-387/U waveguide flange with the alignment, and the screw holes, was simulated using CST Microwave Studio to verify the overall performance. This antenna finds potential applications in future 6G wireless communication systems.

## Structure and design

### Aperture-FSS unit cell

**Figure 1:**
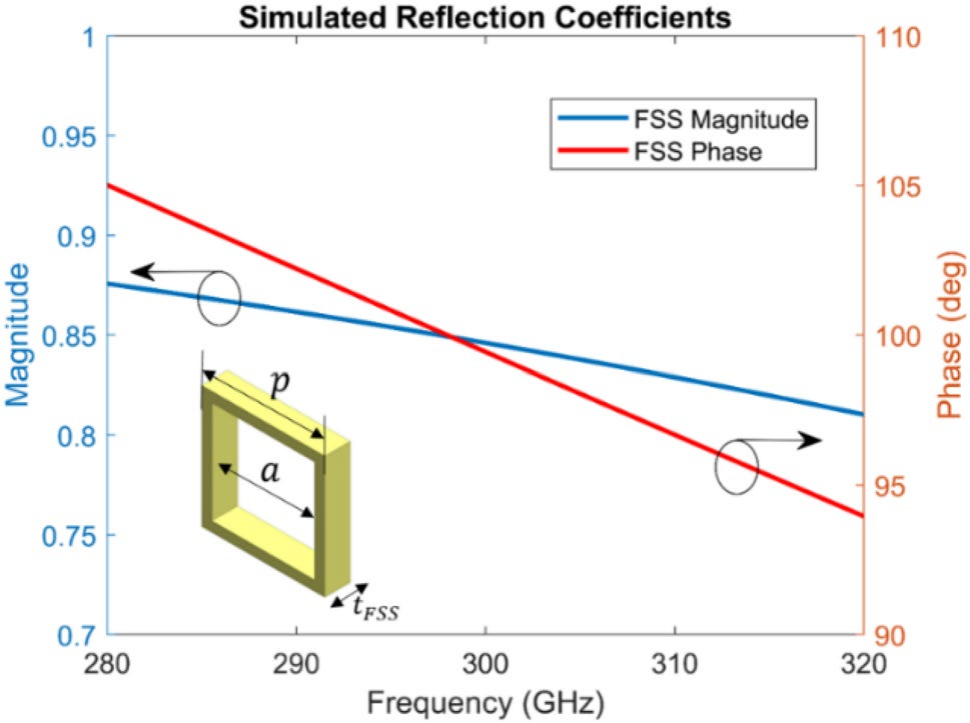
Simulated complex reflection coefficient of the proposed aperture FSS unit cell (dimensions is inset).

In^13^, a PRS is placed at a half-wavelength height above the ground plane to form a one-dimensional cavity, leading to a high directive beam radiation, which is generally explained by the ray-tracing or leaky-wave approach^18,19^. A primary radiator such as a radiating slot is used to excite the cavity. In our proposed antenna, the aperture FSS layer works as PRS, where a square aperture element has been chosen for the design. The geometry of the aperture FSS unit cell is shown in Fig. 1. The optimized dimensions of each aperture ‘$a$’ are 0.42 ${\lambda }_{0}$× 0.42 ${\lambda }_{0}$ and the periodicity ‘$p$’ is 0.5 ${\lambda }_{0}$ as shown in Fig. 1. The thickness of the FSS layer is 0.1 ${\lambda }_{0}$.

The initial simulations were performed on the unit-cell structure with periodic boundary conditions for computational efficiency. The simulated reflection characteristics (magnitude and phase) of the aperture FSS unit cell is shown in Fig. 1. High values for the reflection coefficient magnitude are achieved for the unit cell with dimensions inset in Fig. 1, indicating high antenna directivity. The aperture FSS unit cell exhibited a positive reflection phase gradient over frequency, which resulted in improved antenna bandwidth^13,20^.

### High-gain sub-THz FPC antenna with aperture-FSS layer

**Figure 2:**
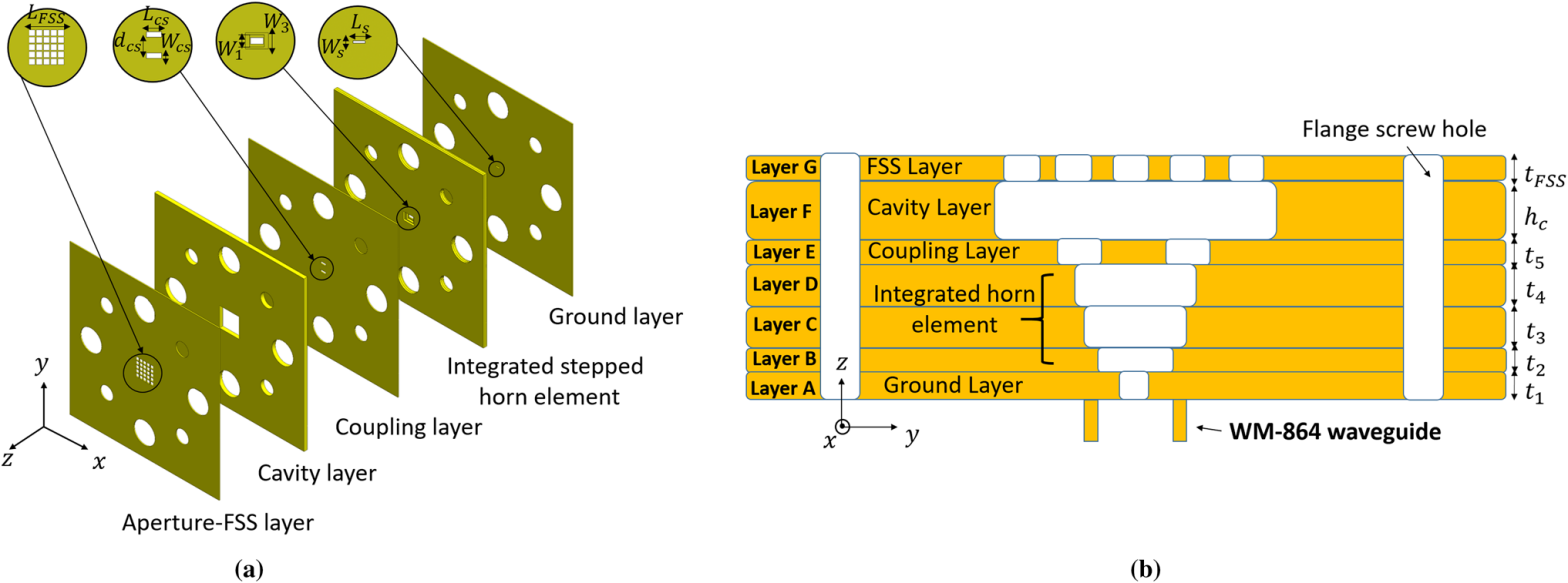
Configuration of the proposed 300 GHz FPC antenna with holes of standard WM-860 waveguide flange. (**a**) 3D-explosive structure (**b**) and sectional side-view.

The configuration of the proposed sub-THz linear polarized FPC antenna is illustrated in Fig. 2. It consists of seven metallic layers, namely the ground layer, the integrated stepped horn element (three layers), the coupling layer, the cavity layer, the aperture-FSS layer, from the bottom to the top. The ground layer (layer A), a slot antenna works as a primary radiator with broadside radiation, has a dimension of ${L}_{s}=0.48 {\lambda }_{0}$ and ${W}_{s}=0.1 {\lambda }_{0}$ and thickness of ${t}_{1}=0.1 {\lambda }_{0}$; where ${\lambda }_{0}$ is the free space wavelength at 300 GHz. A slot-fed waveguide has been used as the primary radiator for the whole antenna structure. The waveguide is a standard WM-864 rectangular one with a UG-387/U flange, works from 220 to 330 GHz.

Maintaining high gain over a wide bandwidth is a big challenge for such FPC antenna design, and as a result, the horn element is integrated. The three layers (B, C, and D) are employed to construct the integrated step-profiled horn element as shown in Fig. 2b. The rectangular apertures shape of the horn element has dimensions, as illustrated in Table 1. This stepped horn antenna is used to improve the impedance matching performance of the whole antenna structure^21^. The coupling layer (layer E), comprises two slots that are excited with equal phase and amplitude by the WM-864 waveguide through a ground layer and a stepped horn antenna element. The distance between two coupling slots along *y*-direction is ‘${d}_{cs}$’, which is equal to 0.97 ${\lambda }_{0}$. The cavity layer (layer F), which is supported by a metallic plate shown in Fig. 2a, determines the resonant height ‘${h}_{c}$’ between the feed antenna (i.e. A–E layers) and aperture-FSS layer, which could be calculated by the Eq. (1). The length, width and thickness of this square cavity are ${L}_{c}$, ${W}_{c}$, and ${h}_{c}$, respectively shown in Table 1. The aperture-FSS layer (layer G), consisting of an array of the aperture with a periodicity of ‘$p$’ between each aperture element. The geometrical parameters of the square apertures in the FSS layer are adjusted to alter the magnitude and phase of incident electromagnetic fields, to achieve highly directivity radiation patterns performance as explained in the above section. The aperture-FSS layer works in a standing-wave environment (i.e., inside a resonant cavity). Fabry–Perot (FP) resonance condition^13^ must be satisfied and is determined by the following equation:

1 $${\varphi }_{FSS}+ {\varphi }_{Co}- \frac{4\pi {h}_{c}}{{\lambda }_{0}}= 2\pi N ,\quad N=0,1,2\dots$$

where ${{\upvarphi }}_{FSS}$ and ${{\upvarphi }}_{Co}$ are the reflection phases of aperture-FSS and coupling layers, respectively. ${h}_{c}$ is resonant cavity height, and N represents the resonance mode number of the FP resonant cavity. Only the zeroth-order mode (N = 0) of the FP cavity is considered to keep the low-profile of the antenna. When the resonant condition is satisfied, the maximum directivity at the broadside is obtained^22^.

**Table 1:** Antenna design parameters (Units: mm).

Layers	Parameters	Value	Parameters	value	Parameters	value
Ground	${t}_{1}$	0.1	${L}_{s}$	0.48	${w}_{s}$	0.1
Integrated horn element	${t}_{2}$	0.1	${L}_{1}$	0.8	${w}_{1}$	0.4
	${t}_{3}$	0.2	${L}_{2}$	1	${w}_{2}$	0.75
	${t}_{4}$	0.2	${L}_{3}$	1.42	${w}_{3}$	1
Coupling	${t}_{5}$	0.1	${L}_{cs}$	0.7	${w}_{cs}$	0.25
Cavity	${h}_{c}$	0.44	${L}_{c}$	2.6	${w}_{c}$	2.6
Aperture-FSS	${t}_{FSS}$	0.1	${L}_{FSS}$	2.3	${w}_{FSS}$	2.3

**Figure 3:**
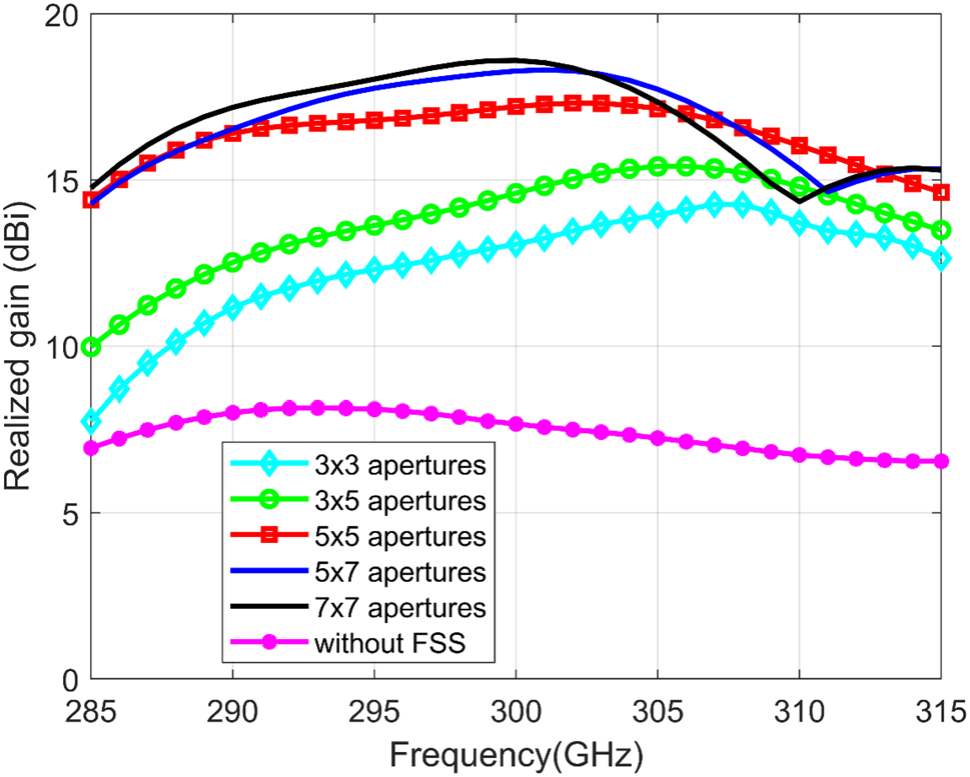
Realized gains of the 300 GHz FPC antenna with different aperture-FSS configurations.

In order to analyze the performance of the FPC antenna based on aperture-FSS layer, keeping all other parameters fixed, realized gains of the proposed antenna with different aperture-FSS configurations are shown in Fig. 3. As can be observed in the figure, when there is no aperture-FSS layer above the cavity, the antenna gain is around 7.5 dBi. By introducing the proposed aperture-FSS layer, the antenna gain can be improved significantly when compared with without FSS layer. The optimum lateral size of the FSS layer was 2.32 ${\lambda }_{0}\times$ 2.32 ${\lambda }_{0}$ at 300 GHz, which equates to 5 × 5 aperture-FSS unit cells. This size was chosen to give a best moderate size in terms of fabrication with stability gain over desired bandwidth. The peak realized gain of 17.2 dBi is obtained around 300 GHz. It increases as expected by increasing the FSS area. The effect of FSS area on the return loss (S11) performance was found to be negligible.

**Figure 4:**
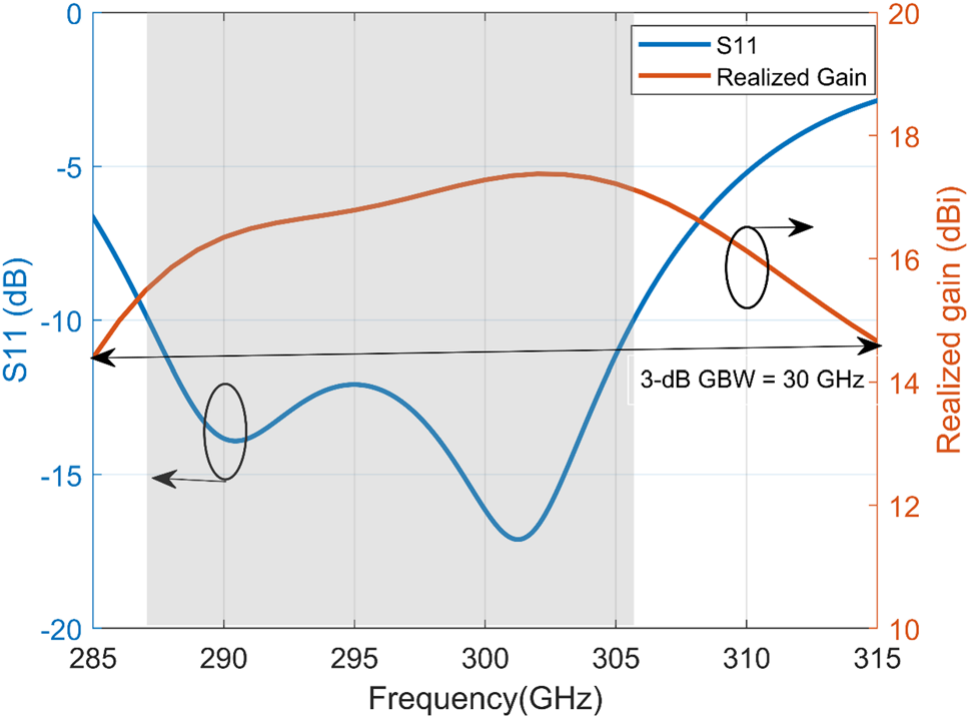
Simulated reflection coefficient (S11) and realized gain of the proposed antenna.

Figure 4 illustrates the performances of the proposed sub-THz FPC antenna with standard UG-387/U waveguide flange. As can be seen, the impedance bandwidth covers from 287 to 305.5 GHz with a bandwidth of 18.5 GHz for the reflection coefficient ≤ −10 dB. The simulated maximum directivity obtained for the proposed aperture-FSS FPC antenna at broadside is 17.87 dBi (i.e., 61.24). The 3-dB gain bandwidth (3-dB GBW) calculated from the results in Fig. 4 is 30 GHz (10%). Based on these results, a figure of merit for the aperture-FSS FPC antenna defined by the product of maximum directivity and the 3-dB broadside gain bandwidth is (61.24) × (0.1) = 6.124, which is close to the value of 7.11 for a thick PRS^23^ reported at 60 GHz. The directivity-bandwidth figure of merit is 2.48 times larger than the best obtainable with planar and thin PRS structures^10^.

**Figure 5:**
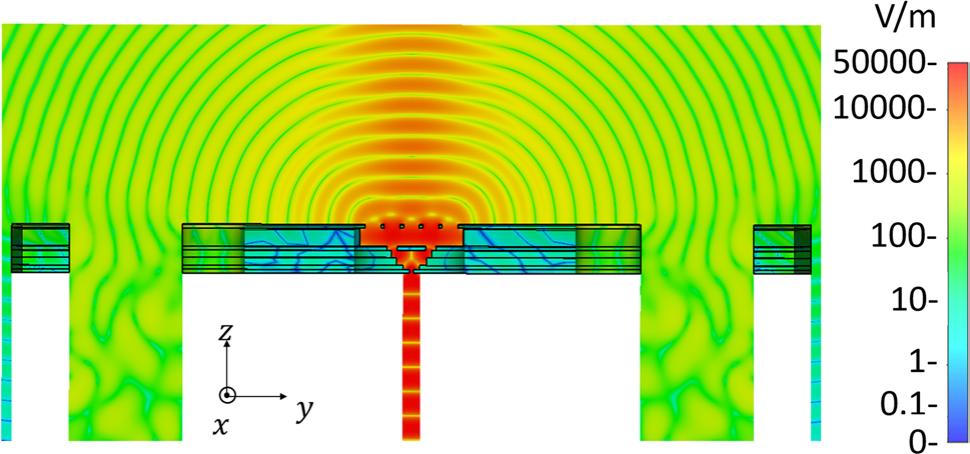
Simulated E-field distributions of the proposed antenna with standard UG-387/U rectangular flange holes at 300 GHz.

Figure 5 gives the E-field distributions of the whole antenna with aperture-FSS layer including holes of alignments pins and fixated screws for standard UG-387/U rectangular flange at 300 GHz. We note that; the EM energy converges along the boresight direction.

### Fabrication technology

**Figure 6:**
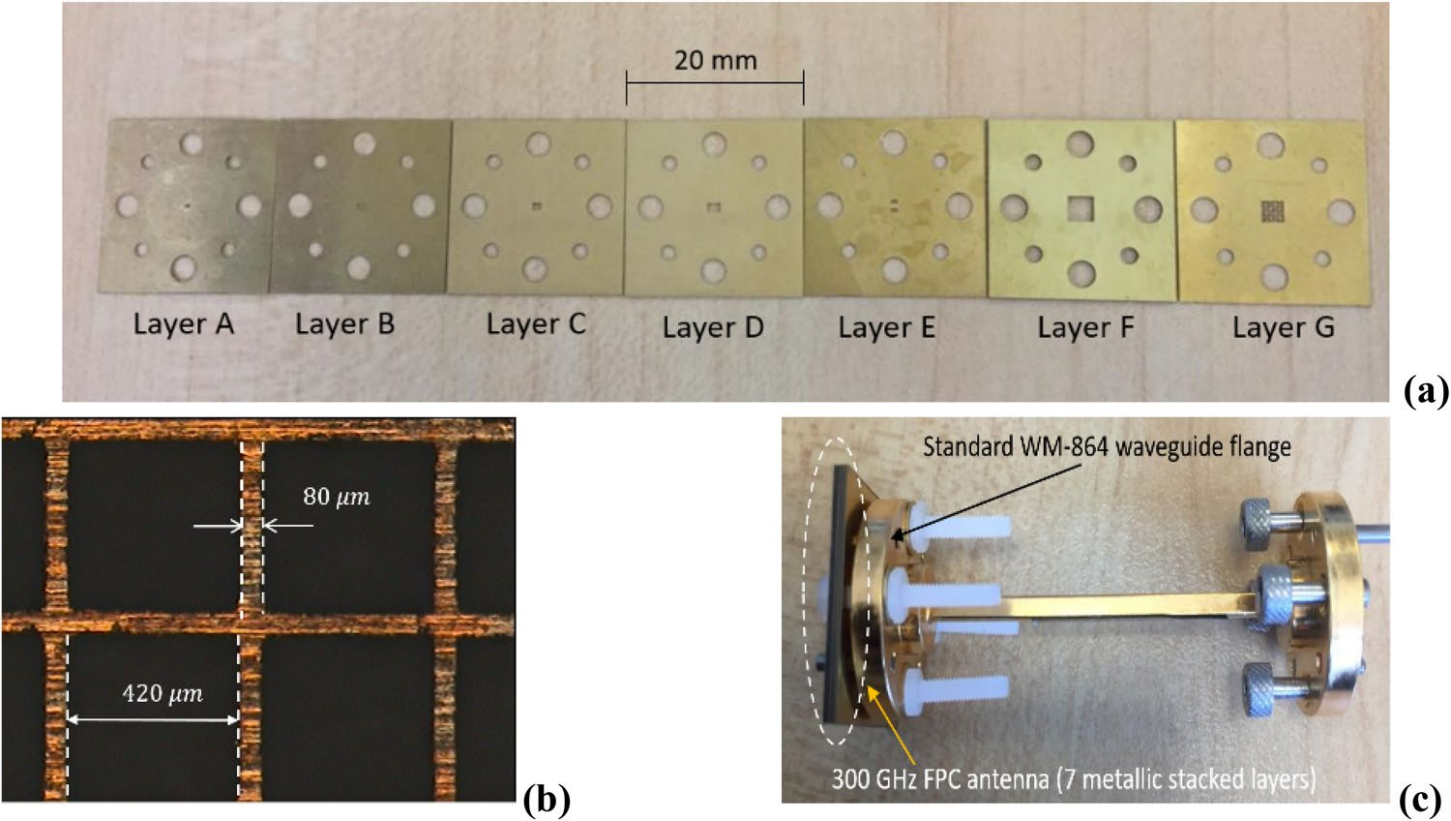
Photographs (**a**) 7 brass metal layers required to assemble one antenna, (**b**) microscope image of the aperture-type FSS layer and, (**c**) the manufactured antenna mounted on a standard WM-864 waveguide flange. The antenna is aligned using two standard alignment pins and fixated with four plastic screws. The standard flange size is 20 mm × 20 mm.

A laser cutting brass technology has been used for each metal layer in the proposed antenna using LPKF ProtoLaser U4 laser machine with technical support from M2ARS (Ch. Guitton and F. Boutet). The seven brass metal layers needed for one antenna assembly, having different thicknesses as shown in Table 1, have been used to manufacture the proposed 300 GHz FPC antenna are shown in Fig. 6a. This brass is often used as a laser-cut metal, which is a highly reflective material. All brass metal layers are fixed by using four plastic screws as shown in Fig. 6. The ultraviolet (UV) laser beam wavelength (λ = 355 nm in the UV spectrum), is focused on each brass metal layer individually having a different thickness to obtain the desired dimension, with the appropriate settings, such as laser cutting speed of 200 mm/s, and a laser spot size of 20 µm. The proposed antenna with aperture-FSS layer is fabricated and assembled, which is shown in Fig. 6. The complete antenna comprises a feed antenna part (A–F layers), a 5 × 5 metallic aperture-FSS part (G layer), and a standard WM-864 rectangular waveguide with UG-387/U flange. The metal layers contain holes for the alignment dowel pins and screws, which enable a direct connection to the standard UG-387 waveguide flange without any additional test fixtures or interfaces. This direct-mount technique is superior to alternative setups using silicon-micromachining without bonding the alignment method^24^. We can notify that the laser cutting technology uses only brass metal layers, without the need of a metallization process. But silicon micromachining uses dielectric substrate and it is metallized, the thickness of metallization must be more than two or three times of the skin depth in order to reduce the ohmic losses at higher frequencies. Another significant point is the need for a mask, and clean room for silicon micromachining which are more expensive but not the case for laser-cutting technology. On the other hand, silicon micromachining provides more accuracy. Also, for silicon micromachining we are limited to a thickness of 400 μm accuracy but for laser-cutting we succeeded to reach 800 μm thickness accuracy.

## Experimental results and discussion

The reflection coefficient characterization (S11) is measured using a Rohde &Schwarz ZVA67 vector network analyzer (VNA) and a Virginia Diodes Inc. (VDI) frequency extender module (220–330 GHz) with WM-864 waveguide flange interface. Figure 6b shows a microscope image of the aperture-type FSS layer, where the actual sidewall width was found to be 80 μm. Figure 6c shows the manufactured antenna mounted in a standard WM-864 waveguide flange.

**Figure 7:**
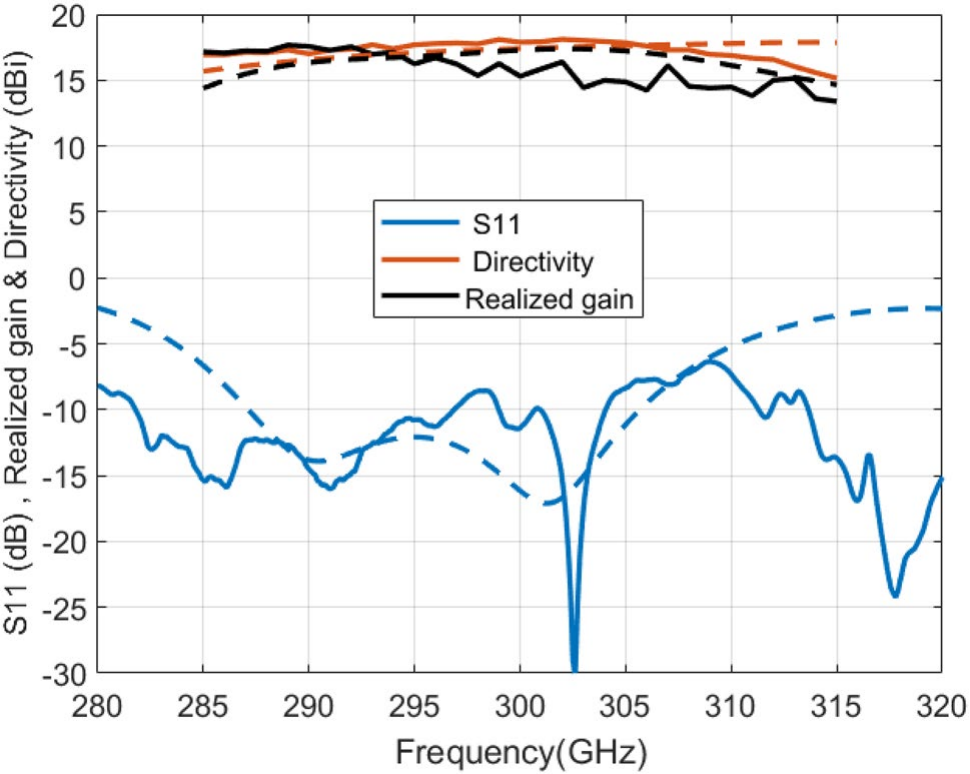
Measured (solid lines) and simulated (dashed lines) of (S11), directivity and realized gain for 300 GHz FPC antenna prototype.

The measured reflection coefficient (S11) is below − 10 dB at the working band from 282 to 304 GHz with a bandwidth of 22 GHz is shown in Fig. 7. Figure 7 also indicates the measured realized gain of the antenna, and it shows that the gain ranges from 14.4 to 17.7 dBi in the frequency band of 285–310 GHz. There is some divergence between the measured and simulated realized gain; this may be because of assembling and fabrication tolerances that are normal in the 300 GHz band. The measured and simulated directivities of the proposed antenna is also presented in Fig. 7.

To demonstrate the characteristics of the proposed antenna, such as the radiation patterns, are measured in a compact antenna test range (CATR) chamber at IETR (funded by the European Union through the European Regional Development Fund, through the CPER Projects 2015–2020 SOPHIE/STIC and Ondes).

**Figure 8:**
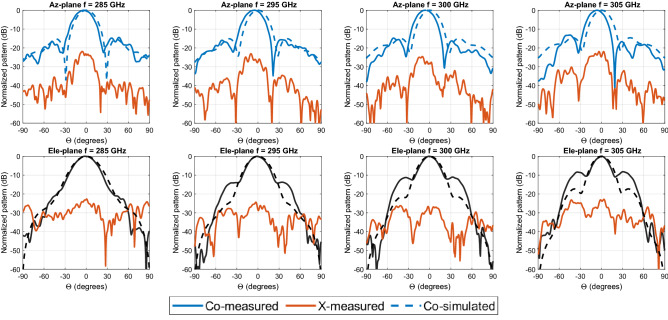
Measured (solid lines) and simulated (dashed lines) normalized radiation patterns of the proposed antenna of the azimuth (Az.) and elevation (Ele.) planes at different frequencies.

**Figure 9:**
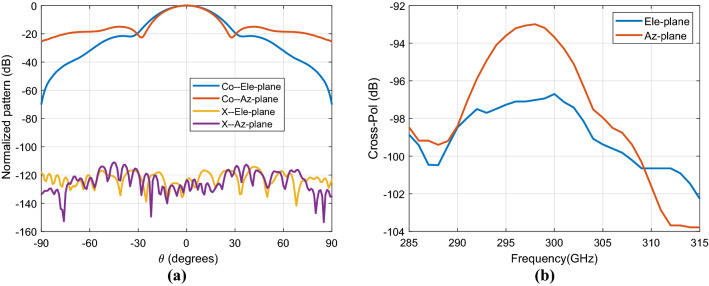
Simulated (**a**) normalized cross-pol radiation patterns at 300 GHz and (**b**) cross-pol level of the FPC antenna prototype.

The measured elevation (Ele.)-plane and azimuth (Az.)-plane radiation patterns, shown in Fig. 8, are in acceptable agreement with the simulation results. In the measured elevation-plane cuts, the main beam is a broadside direction. Some minor discrepancies have to be noticed on the side lobe level (SLL), and the SLL is kept below 15 dB as designed, except at higher frequency (i.e., 305 GHz). The measured main beam in azimuth-plane cuts is a slight shift from broadside direction; this is because the flange used has a small tilt (the long waveguide section in photo, Fig. 6c), the SLL is kept below 15 dB as designed. The measured cross-polarization levels are more than 25 dB at these frequencies. The simulated cross-polarization in both principal planes is more than 93 dB over the entire bandwidth of interest. The cross-polarization radiation pattern for one of the frequencies at 300 GHz is presented in Fig. 9a.The maximum cross-polarization level at broadside direction is less than − 93 dB over the whole desired band as shown in Fig. 9b.

**Figure 10:**
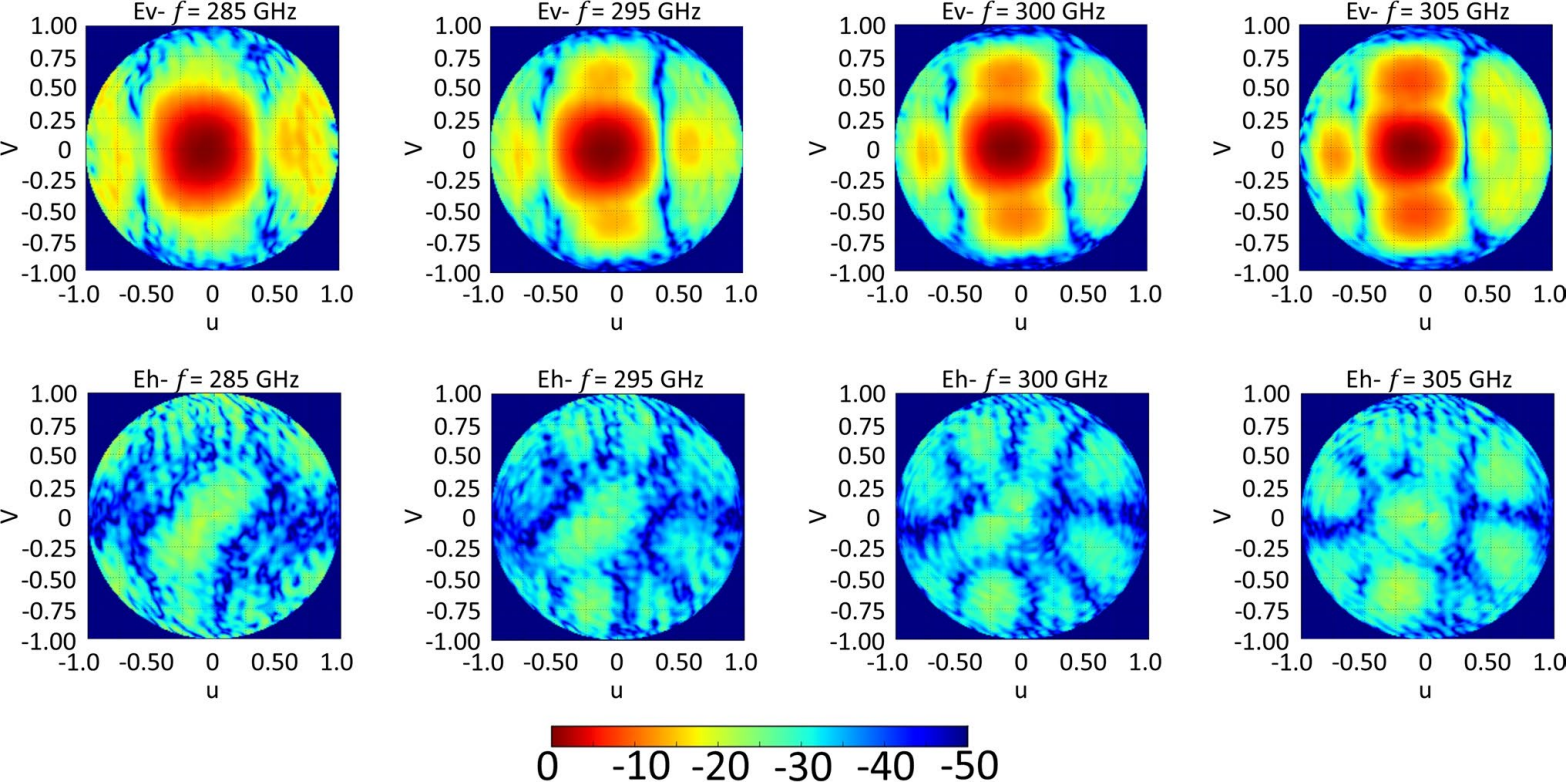
Measured 3D normalized radiation pattern of the proposed antenna (Co ‘Ev’ & Cross ‘Eh’—polarization components), in the u–v spectral plane at different frequencies. The color bar is on the dB scale.

Figure 10 shows the measured 3D normalized radiation patterns of the proposed antenna in the *u*–*v* plane coordinate system ($u= \mathrm{sin}\theta \mathrm {cos}\phi$, and $v= \mathrm{sin}\theta \mathrm{sin}\phi$) at different frequencies, to verify the results and investigate the radiation characteristics outside the two principal planes, where the advantage of transformation to the u–v plane is evident as well. The measured radiation pattern shows a highly directive pattern in all plane cuts, which coincides with the simulation results.

Table 2 compares the performances of proposed fully metallic FPC antenna at 300 GHz with different sub-THz reported prototypes in the literature. It can be seen that the proposed antenna has a low-profile planar structure using the Fabry–Perot resonant cavity without any dielectric materials. The proposed antenna is fabricated using low-cost laser cutting technology, and also achieves higher gain and good wide bandwidth as compared to those using different high-cost technologies. These facts indicate that the proposed antenna structure is a good candidate in sub-THz applications.

**Table 2:** Key factor comparison of measurement of this work and other published works.

References	Antenna type	Manufacturing process	Center frequency (GHz)	Bandwidth (GHz)	Gain (dBi)	Size (mm^3^)	Fabrication complexity
^25^	Profiled corrugated horn	LTCC	300	100	18	5 × 5 × 2.8	High
^17^	Fabry–Perot cavity	Micromachining	284	~ 4.5	15.9	11.8 ×11.8 × 8.45	Low
^6^	Quasi-planar reflectors	Metallic milling	400	175	> 26.5	16 ×16 × 5.5	High
This work	Fabry–Perot cavity	Laser cutting brass	300	22	17.7	2.6 × 2.6 × 1.24	Low

## Conclusions

A linearly polarized sub-THz FPC antenna with high gain and low cross-polarization has been presented. The proposed antenna has been designed with standard WM-864 rectangular waveguide flange, and it has been fabricated in brass metal using low-cost laser-cutting technology. This laser cutting brass technique has been shown to provide the fabrication of complicated details of the design with high precision. The 300 GHz FPC antenna has been measured using facilities at IETR, and the results obtained are in good agreement with simulation results.
